# Intensivist and COVID-19 in the United States of America: a narrative review of clinical roles, current workforce, and future direction

**DOI:** 10.11604/pamj.2022.41.210.29956

**Published:** 2022-03-14

**Authors:** Nadia Nazir Jatoi, Sana Awan, Maham Abbasi, Momina Mariam Marufi, Muhammad Ahmed, Shehzeen Fatima Memon, Nimra Farooqui, Maaz Hasan Khan, Hadi Saiyid, Abdurrahman Husain, Kaneez Fatima, Shahram Maroof, Atul Malhotra

**Affiliations:** 1 Department of Internal Medicine, Dow University of Health Sciences, Karachi, Pakistan,; 2 Department of Internal Medicine, Jinnah Sindh Medical University, Karachi, Pakistan,; 3 Department of Internal Medicine, United Medical and Dental College, Karachi, Pakistan,; 4 Department of Medicine, Division of Pulmonary, Critical Care and Sleep Medicine, University of California San Diego, San Diego, United States of America,; 5 Pulmonary and Critical Care, John H Stroger, Hospital of Cook County, Chicago, United States of America

**Keywords:** Healthcare workers, personal protective equipment, intensive care unit, COVID-19, World Health Organization

## Abstract

COVID-19 continues to spread across borders and has proven to be a challenge for the existing healthcare system. The demand for intensivists has dramatically increased in the United States, in the backdrop of an expected lack of intensivists in many States even before the pandemic. One proposal has been to organize multidisciplinary teams functioning under one intensivist, as this approach would make use of the existing healthcare force and lessen the burden on intensivists. Another recommendation is the adaptation of Tele-ICUs, which have demonstrated constructive outcomes in the past. Moreover, ensuring the provision of all types of personal protective equipment, adequate testing and, other provisions such as mental health support, financial incentives for intensivists should be prioritized. More intensivists should be trained for the future, for which better institutional policies are essential.

## Introduction

On March 11, 2020, the World Health Organization (WHO) announced the severe acute respiratory syndrome coronavirus (SARS-CoV-2) pandemic [1], barreling through 114 countries in three months and infecting over 118,000 people. The first case was reported in Wuhan, China, on December 31, 2019, whereas the first case in the United States was reported on January 22, 2020 [1]. Due to a staggering increase in disease transmission and hospitalization of COVID-19 patients, the most advanced healthcare systems like the United States (U.S) face many challenges [2]. Of note, a great demand is seen for intensivists who care for critically ill patients in the intensive care unit (ICU). Intensivists are physicians trained in their respective primary specialties (for example, internal medicine, anesthesiology, emergency medicine, surgery, and pediatrics) and are also board-certified in critical care medicine [3]. Most intensive care in the U.S is provided under a multidisciplinary umbrella, often involving hospitalists, nurses, respiratory therapists, pharmacists, and advanced practice providers (APP's) [4]. This team is also supported by many other disciplines, including social workers, occupational and physical therapists, and registered dietitians [3]. There are currently fewer than 65,000 physicians, physician assistants, advanced practice nursing intensivists, and about 550,000 critical care nurses in the U.S [1]. Rubenfeld GD *et al*. had previously refuted claims regarding an intensivist shortage in the U.S and had called for alternate solutions to address the issue of providing adequate critical care [5] however, the unexpected emergence of the novel COVID-19 pandemic has unmasked the acute shortage of intensivist, which could leave a long-lasting impact on the U.S healthcare system. A need for intensivists could lead to detrimental effects on patients' health by increasing their length of stay in the ICU, in conjunction with increased complications such as pneumonia, re-intubation, and many other medical errors [6-8]. Therefore, it is crucial to identify the gaps and explore potential options to bridge the gap between the shortage of the intensivist workforce and critical care during this pandemic [9]. In this review, we seek to highlight the role played by the critical care specialists, determine the underlying reasons for the current and past intensivists' attrition in the USA, and propose possible measures that can be taken to mitigate the shortage of intensivists to prepare for any future pandemic.

## Methods

A thorough literature search was done for four months to extract relevant papers for this narrative review study. Research papers in English from 1992 to 2020 were studied from databases such as PubMed, EMBASE, MEDLINE, Google Scholar, Cochrane Library (Wiley), and newspaper media reports. A combination of keywords such as 'intensivist', 'critical-care specialists', critical-care physicians', COVID-19, SARS-CoV', 'COVID-19 pandemic', 'workforce shortage', 'intensivist shortfall', 'intensivist shortage' was searched. The extracted papers were then scrutinized for relevance to the review topic, and subsequently, papers pertinent to the topic were included. 67 papers were read for final review writing after discarding 20 irrelevant papers.

## Current status of knowledge

### Roles played by intensivists in managing COVID-19 patients

**Leading from the front in intensive care units (ICUs):** intensivists mainly act as the leaders of an ICU team [10]. They actively manage respiratory failure, including providing non-invasive or invasive ventilation, hemodynamic instability, and multi-organ failure requiring advanced support such as continuous renal replacement therapy in COVID-19 patients. The initial protocol requires triaging to accommodate patients likely to gain the maximum advantage from ICU. ICU practitioners must identify COVID-19 patients presenting with confirmed or suspected acute respiratory distress syndrome (ARDS) [11]. It is crucial that while dealing with high-risk patients, healthcare workers are protected utilizing appropriate personal protective equipment (PPE) at all times. Repeated testing is an integral component of accurately labeling the patient COVID-19 positive [11].

**Management of complications in COVID-19 patients:** a book issued by a joint effort of pulmonologists from different parts of the world, International Pulmonologist's Consensus on COVID-19, gives a detailed approach to dealing with critically ill patients in ICU. As respiratory complications are the most likely reason for ICU admissions, pulmonologists dealing with patients with acute respiratory distress syndrome (ARDS) need to monitor for worsening conditions closely. If a patient's condition deteriorates, it should be taken with extreme precaution as many require intubation which poses a significant threat for spreading the infection [12]. As a COVID-19 complication, myocardial injury, owing to increased cytokine storm, has been noted, and the cause of death is an imminent cardiac shock in 40% of cases [13]. Moreover, intensivists should provide oversight on timely rehabilitation, including physical therapy, and diligent nutritional support should be adopted to minimize ICU-related muscular breakdown and delirium [14].

**Figure 1:**
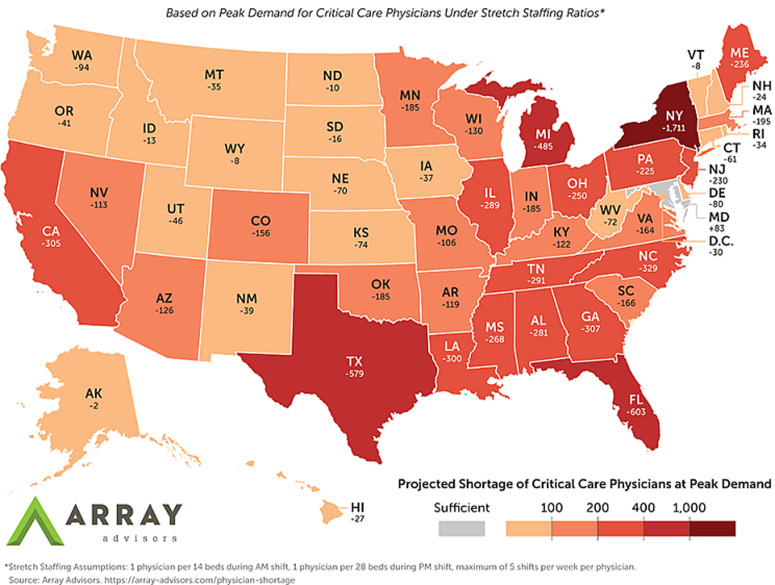
projected critical care physician shortage by State during COVID-19

**Shortage of intensivists in the United States:** the U.S is currently under a continued crisis of shortage of intensivists owing to the COVID-19 pandemic [3]. While the country's primary focus is on the lack of medical supplies, ICU beds, and ventilators, the ICU staffing shortage has emerged as a significant concern [15]. Based on the American Hospital Association (AHA), 48% of the surveyed hospitals were deficient for their critical care staff [16]. As the current pandemic has progressed, urgent care has been mandated for more patients; hence the acute demand for dedicated teams of the intensivist, APPs, and ICU staff has greatly increased [3]. According to the new model published in March 2020, the country is likely to face a crippling shortage of more than 7900 critical care physicians during the current crisis. Apart from Maryland, all States and the District of Columbia are likely to face deficits. The majority of the States will require 50 to 300 additional critical care physicians to meet the expected demand (Figure 1). New York is expected to experience a shortage of over 1,700 critical care physicians, which is more than three times their current capacity [17]. Recruiting retired intensivists to help with the increased demand has been adopted in many places [3]. However, as the risk of infection increases with increasing age, many older nurses and physicians are compelled to stay at home to minimize their risk of exposure. This issue decreases the chances of gathering highly trained staff to help alleviate the current crisis [18]. Despite this concern, a lack of extensive testing has led to many health care workers (HCW's) in the country self-isolating themselves [19]. Increased testing of intensivists might help increase the workforce's capacity by halting the unnecessary self-quarantine measures taken by the HCW's [20].

To eliminate the intensivist shortages during the current pandemic, 31 teams were prepared, including 598 intensivists and 2319 ICU nurses from different cities of the U.S. They were moved to hospitals with the greatest needs since early January 2020 [21]. However, it should be noted that the time taken to acclimatize to new surroundings adds to the delay in efficient teamwork. Moreover, details regarding the epidemiological and clinical characteristics of the virus are still unknown. The differences between individual clinical experiences of these teams can be somewhat conflicting, leading to decreased efficiency of patient care [3]. Even though COVID-19 continues to evolve and threatens the already overwhelmed health systems, another problem does not seem too far off. A slight rise in the number of critically ill children is seen with an emerging disease termed the 'Pediatric Inflammatory Multisystem Syndrome associated with SARS-CoV2 (PIMS-TS)'. First appearing in England, it can now be seen in New York City and elsewhere [22]. According to a cross-sectional study of 46 North American Pediatric Intensive Care Units (PICUs), between March 14 and April 3, 2020, 48 children were admitted to 14 PICUs in the USA [23]. With this emerging syndrome that has a clinical picture consistent with shock, more children may require hospitalization and intensive care, increasing the demand for pediatric critical care specialists and PICU staff. The shortage of intensivists in the USA has not been a recent highlight, but a narrative has been building on this issue for over a decade [24]. Two decades ago, the Committee on Manpower for Pulmonary and Critical Care Societies predicted, in succeeding years, the need to exceed the limited supply of intensivists in the U.S. The committee pointed out that although the elderly population with complicated comorbidities has increased in the USA, there has been no significant increase in the intensivist. According to one report in 2006, it was predicted that the USA would need 4,300 critical care physicians by 2020, and the shortfall will rise to 1,500 physicians [25].

### Reasons behind the shortage of intensivists during the current pandemic

**Lack of personal protective equipment (PPE):** the highly contagious nature of coronavirus and the shortage of PPE augments the already worsened situation of first-line workers. The lack of PPE will subsequently result in healthcare professionals working in high dependency units acquiring infections, causing them to go into isolation. Centers for Disease Control and Prevention (CDC) estimates that more than 60,000 health care workers have been infected, and close to 300 have died from COVID-19 [26]. Inadequate testing kits and lack of PPE increase the risk of infection and, ultimately, the death toll. Steps must be taken to regulate the supply of PPEs as this will help retain a more critical care workforce in the field.

**Lack of financial incentives:** intensivists and other critical care staff should be provided extra compensation if their duties necessitate extended hours along with access to support services [27]. However, due to the financial ramifications of the pandemic, despite shortages of nursing staff in some parts of the country, many nurses are being furloughed [28]. American healthcare companies are looking to make budget cuts since a sizable loss of income is seen due to postponed elective procedures. Before the pandemic, the federal funds for State, local, and tribal public health preparedness were cut from $940 million in 2002 to $675 million in 2019. In contrast, health care emergency preparedness was cut by nearly 50%, from $515 million in 2004 to $265 million in 2019 [29].

**Physician burnout:** COVID-19 has remarkably increased the mental and physical burden on the healthcare workers (HCWs) working on the front line [30-37]. They are not just treating a vast number of critically ill patients daily while witnessing multiple deaths but are also risking the lives of their colleagues and loved ones, in addition to their own. Their mental well-being worsens due to the uncertainty surrounding the entire situation. This situation puts them at an increased risk of burnout due to difficult working hours and inadequate protective equipment while dealing with the patients. In a study conducted in Vancouver, 68% of physicians reported burnout during the current pandemic [36]. Another study found that SARS-related fear was predictive of symptoms of Post-Traumatic Stress Disorder [38]. Institutions such as the University of North Carolina have taken steps to ensure therapeutic support by working on telehealth, keeping flexible schedules, and setting up support hotlines [38]. Further therapy options similar to these in other hospitals are imperative for the well-being of the workers battling against the pandemic.

**Recommendation to adequately address intensivist shortage for the present and future:** after an extensive review of the literature, the following short and long-term measures can be adopted to counter the shortage of intensivists during the current pandemic (Table 1).

**Table 1:** recommendations to manage intensivist shortage for the present and future

Recommendations for the present	Recommendations for the future
Establishing multi-disciplinary teams that include general surgeons, anesthesiologists, APPs, etc	Implementation of tele-ICUs
Recruiting additional workforce including ICU team members such as APPs	Introducing succession-planning into the ICU community
	Increasing fellowship programs and training opportunities
	Devising better policies to attract professionals into critical care

### Recommendations for the present

**Figure 2:**
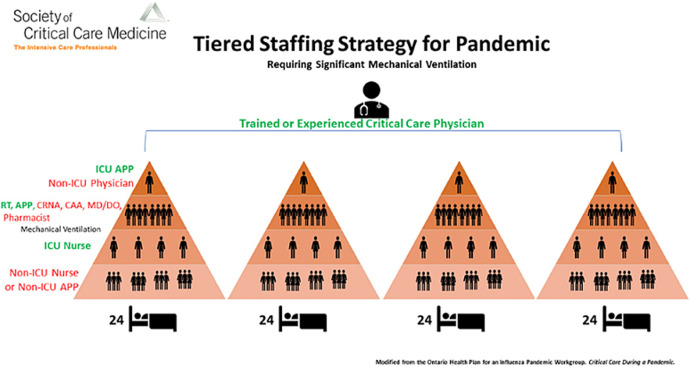
workforce model adopting a tiered staffing strategy to maintain an adequate physician-patient ratio

**Organization of multi-disciplinary teams:** many hospitals have the advantage of having a diverse team of certified specialists in critical care, for example, pulmonologists, trauma surgeons, neonatal ICU specialists, emergency medicine physicians, pediatric ICU specialists, anesthesiologists, etc. These specialists might prove beneficial if cared for and help cover ICU beds as the demand surpasses the existing supply of intensivists. However, this option is most likely feasible at academic medical centers but may be more challenging in some community hospitals [17]. Therefore, the lack of a uniform critical care model has compelled various sub-specialties and critical care societies to propose ideas to counteract the intensivist' shortages in the past years [39]. Similarly, the Society of Critical Care Medicine (SCCM) has endorsed a tiered system to strengthen the battle against HCW shortages during COVID-19. This algorithm has been designed to help benefit patients requiring ventilator support during emergencies (Figure 2). In this proposed system, one physician, experienced in critical care, looks after 4 teams of clinicians with diverse experiences. The 4 teams are composed of 24 patients each, and a physician other than an intensivist, i.e., anesthesiologist, surgeon, etc., is placed on top of each team. These teams are further filled in by advanced practice providers (APPs) and respiratory therapists who assist the non-ICU expert in providing dedicated intensive care. Surgeons and Anaesthesiologists can be explicitly recruited as most elective procedures are usually delayed on a large scale due to COVID-19 [3].

**Recruitment of additional workforce:** many authors and the Society of Critical Care Medicine (SCCM) have suggested the increased utilization of APP's during the coronavirus crisis to highly trained intensivists because of their extensive knowledge of ICU-dedicated care [17]. In some States, such as Florida, laws have been passed to allow nurse practitioners to care for patients independently [17]. Furthermore, we recommend that intensivists who are currently in their training years, i.e fellowship or residencies, be allowed to work. Waiving U.S licensing rules for the provision of international Tele-ICU assistance might also prove beneficial.

**Tele-ICUs:** tele-ICU is a command center in which technology connects a critical care team to the critical care ward [40]. Tele-ICU is fundamentally designed to help nurses and doctors manage multiple units and patients in hospitals and remote locations. Checking vitals are as easy in Tele-ICUs as is at bedsides [41]. Studies have shown decreased mortality rates and length of stay in the ICUs after adapting to Tele-ICU [42]. Wherever transport issues discourage the movement of staff, Tele-ICU makes it possible to provide critical care to reach remote areas. All patients being supervised using Tele-ICUs are re-accessed every 1-4 hours but can also be demand specific. Perhaps the more significant advantage is that all details are quickly submitted by Intensivist Electronic Medical Records systems that operate and augment Tele-ICUs, allowing patient follow-up and management much more manageable. However, teamwork is crucial to ensure smooth delivery of standard treatment and enhanced care of all patients [41-43].

### Recommendations for the future

**Succession planning in the critical-care healthcare system:** as most of the critical care management teams are led by a single leader, succession planning would greatly benefit the ICU setting in preparing for the current and future pandemics [44]. Succession planning not only ensures the preservation of a limited workforce but also helps fill vacant positions efficiently and helps prevent the ICUs from complicated situations caused by attrition. This goal is mainly achieved by prioritizing training and development methods. Many leaders have previously restricted themselves from sharing experiences, but succession planning is gaining popularity and is now deemed necessary [45]. However, it primarily revolves around leadership development and good training processes to utilize talents [46].

**Increasing fellowship training and opportunities:** just like how increasing fellowship programs answered the concerns for shortages of intensivists in 2000, the current situation must help devise ways to increase post-graduate training programs in critical care in the country [47]. Although there has been an increase in fellowship programs for critical care in the last 16 years [47], the demand and supply chain continues to be strained. At present, there are 5 specialty critical care fellowship programs approved by accreditation council for graduate medical education (ACGME) and include anaesthesiology, pulmonology, general surgery, internal medicine, and paediatrics. The number of fellowship programs and residents varies significantly between these specialties [47].

**Adopting artificial intelligence:** artificial intelligence (AI) could be introduced into hospitals to complement existing ICU personnel [17]. Using computers to facilitate the treatment of ICU patients is not a new concept. Previously, their usage has been advised to monitor oxygen, manage patients on ventilators, and care for those with acute respiratory distress syndrome. Artificial intelligence computers are said to learn the information in addition to performing instructed tasks. Artificial intelligence helps intensivists deal with information overload and construct an autonomous ICU with self-monitoring capacities if intelligent machine learning monitors are introduced [48].

## Conclusion

With the increased surge in critical patients requiring intensive care unit (ICU) treatments during the COVID-19 pandemic, the need for intensivists and other respective critical care HCWs has greatly increased. The shortages of intensivists are particularly of great concern as they are recognized as leaders of the ICU teams. Better policies must be devised at the institutional and governmental levels to ensure the extensive availability of intensivists in most American hospitals. However, previously validated strategies to address the current workforce shortage during the current pandemic, if implemented, can significantly minimize intensivist' attrition. Moreover, it should be appreciated that the need for ICU APPs and other critical care dedicated clinical staff will continue to rise in the following years. The provision of PPEs, financial incentives, and mental health support should also be considered to encourage the existing workforce and boost their morale. The situation calls for an immediate and efficient response from authorities at both State and national levels.

### What is known about this topic

- Evidence of the pivotal role of intensivists in the healthcare setting and their shortage in the United States;
- The increased demand for intensivists is owing to the complications brought about by COVID-19;
- Evidence of increased risk of infection and mental burnout due to COVID-19 amongst frontline healthcare workers

### What this study adds

- Identifies the reasons for the scarcity of critical care workforce in the ICU setting during the COVID-19 pandemic, such as the lack of PPE and financial incentives;
- Suggests short-term measures that can potentially mitigate the impact of a shortage of intensivists, such as the organization of multi-disciplinary teams, increased utilization of APPs, and succession planning in the ICU setting;
- Explores the future possibility of utilizing technology such as Tele-ICU and artificial intelligence during the current and future pandemics to ensure well-staffed critical care units during the current and future pandemics.
